# Systemic Growth of *F. graminearum* in Wheat Plants and Related Accumulation of Deoxynivalenol

**DOI:** 10.3390/toxins6041308

**Published:** 2014-04-10

**Authors:** Antonio Moretti, Giuseppe Panzarini, Stefania Somma, Claudio Campagna, Stefano Ravaglia, Antonio F. Logrieco, Michele Solfrizzo

**Affiliations:** 1 Institute of Sciences of Food Production, National Research Council (ISPA-CNR), Via Amendola 122/O, Bari 70126, Italy; E-Mails: giuseppe.panzarini@ispa.cnr.it (G.P.); stefania.somma@ispa.cnr.it (S.S.); antonio.logrieco@ispa.cnr.it (A.F.L.); michele.solfrizzo@ispa.cnr.it (M.S.); 2 Syngenta Crop Protection Italia, Via Gallarate 139, Milano 20151, Italy; E-Mail: claudio.campagna@syngenta.com; 3 S.I.S.—Società Italiana Sementi spa, Via Mirandola 5, San Lazzaro di Savena 40068 (BO), Italy; E-Mail: s.ravaglia@sisonweb.com

**Keywords:** wheat, *Fusarium graminearum*, seed transmission, systemic growth, deoxynivalenol, deoxynivalenol-translocation, deoxynivalenol-3-glucoside

## Abstract

*Fusarium* head blight (FHB) is an important disease of wheat worldwide caused mainly by *Fusarium graminearum* (syn. *Gibberella zeae*). This fungus can be highly aggressive and can produce several mycotoxins such as deoxynivalenol (DON), a well known harmful metabolite for humans, animals, and plants. The fungus can survive overwinter on wheat residues and on the soil, and can usually attack the wheat plant at their point of flowering, being able to infect the heads and to contaminate the kernels at the maturity. Contaminated kernels can be sometimes used as seeds for the cultivation of the following year. Poor knowledge on the ability of the strains of *F. graminearum* occurring on wheat seeds to be transmitted to the plant and to contribute to the final DON contamination of kernels is available. Therefore, this study had the goals of evaluating: (a) the capability of *F. graminearum* causing FHB of wheat to be transmitted from the seeds or soil to the kernels at maturity and the progress of the fungus within the plant at different growth stages; (b) the levels of DON contamination in both plant tissues and kernels. The study has been carried out for two years in a climatic chamber. The *F. gramineraum* strain selected for the inoculation was followed within the plant by using Vegetative Compatibility technique, and quantified by Real-Time PCR. Chemical analyses of DON were carried out by using immunoaffinity cleanup and HPLC/UV/DAD. The study showed that *F. graminearum* originated from seeds or soil can grow systemically in the plant tissues, with the exception of kernels and heads. There seems to be a barrier that inhibits the colonization of the heads by the fungus. High levels of DON and *F. graminearum* were found in crowns, stems, and straw, whereas low levels of DON and no detectable levels of *F. graminearum* were found in both heads and kernels. Finally, in all parts of the plant (heads, crowns, and stems at milk and vitreous ripening stages, and straw at vitreous ripening), also the accumulation of significant quantities of DON-3-glucoside (DON-3G), a product of DON glycosylation, was detected, with decreasing levels in straw, crown, stems and kernels. The presence of DON and DON-3G in heads and kernels without the occurrence of *F. graminearum* may be explained by their water solubility that could facilitate their translocation from stem to heads and kernels. The presence of DON-3G at levels 23 times higher than DON in the heads at milk stage without the occurrence of *F. graminearum* may indicate that an active glycosylation of DON also occurs in the head tissues. Finally, the high levels of DON accumulated in straws are worrisome since they represent additional sources of mycotoxin for livestock.

## 1. Introduction

*Fusarium graminearum* *sensu stricto* Schwabe [syn. *Gibberella zeae* (Schwein.) Petch] is a worldwide recognized destructive pathogen, agent of Fusarium Head Blight (FHB), one of the most important fungal diseases associated with wheat, worldwide [1]. The direct damages of the diseases associated to *F. graminearum* (yield losses, poor quality of grains, costs for disease management) and the mycotoxin contamination of the raw grains and processed wheat products are the main concerns associated to wheat production and consumption [2]. In particular, *F. graminearum* causes the accumulation of deoxynivalenol (DON) and its acetylated derivatives [3]. Deoxynivalenol is a very toxic compound and represents a serious risk for livestock and human health since DON causes skin irritability, haemorragic syndrome, feed refusal, vomiting, being a strong inhibitor of proteic synthesis [3]. The DON contamination in wheat is regulated by the European Commission with maximum levels fixed at 1.75 µg/g of dried matter for unprocessed durum wheat and 1.25 µg/g for soft wheat [4]. However, since DON can also be transformed in the wheat plant in DON-3-glucoside (DON-3G), a less toxic compound defined also as “masked” DON [5], the possible co-occurrence also of this latter metabolite should be taken into account, when mycotoxin risk for wheat is evaluated. 

The site of primary infection of *F. graminearum* is thought to be the extruded anthers during wheat anthesis (flowering). The fungus infects the wheat spikes when the ascospores and macroconidia land on susceptible wheat heads and enter the plant mostly through natural openings such as stomates. From the infected floret, the fungus can grow through the rachis and causes severe damage in a short period of time under favorable conditions. Kernels that are colonized by the pathogen during late kernel development may not appear to be affected, but may still be contaminated with DON. Infected kernels may be used as seeds for a subsequent wheat crop. These infected seeds, if left untreated, may give rise to blighted seedlings. Moreover, the possibility that *F. gramineaum* colonization of seeds can be transmitted to the whole wheat plants has been reported by several authors [6,7,8]. However, if such colonization could be also extended to the heads by the strains of *F. gramineaum* originated from seeds has controversially been reported. No systemic colonisation of roots, culms, leaves or heads of plants grown from *F. graminearum* infected seeds was observed by Boshoff *et al.* [6]. On the other hand, the presence of *F. graminearum*, monitored in different parts of the wheat plants after inoculation of the stem base, was detected also in heads and flag-leaf node by Mudge *et al.* [7]. The ability of *F. graminearum* to colonize wheat heads was demonstrated also by Poels *et al.* [8], that reported the migration of *F. graminearum* from the infected seeds to the heads. Finally, the final DON contamination of heads due to the production of *F. graminearum* strains originated from seeds or stem base is poorly documented. Therefore the aims of this work were:

1. to evaluate the possibility of seed transmission of *F. graminearum* to the whole wheat plants;
2. to evaluate the DON content in the heads due to its production by *F. graminearum* strains occurring in seeds;
3. to evaluate the amount of possible DON-3G occurring *in planta* due to this type of colonization by *F. graminearum*.

## 2. Results and Discussion

### 2.1. Fusarium and DON Contamination of Kernels Used for the Experiments and DON Production by Strain ITEM 126

The sample of 200 kernels representing the seeds used for the experiments did not show any *Fusarium* contamination after traditional and molecular analyses. No DON was detected in the kernels examined.

The *in vitro* culture of *F. graminearum* strain ITEM 126 produced DON.

### 2.2. Detection of F. graminearum in the Wheat Plants

**Figure 1:**
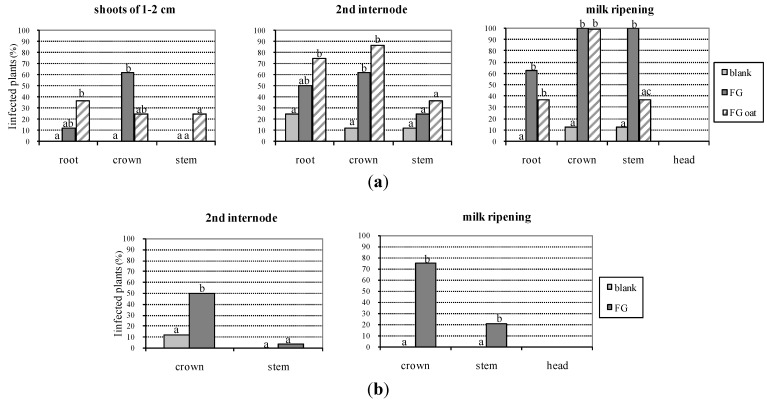
Fungal contamination of portions of the wheat plants evaluated by morphological analysis, shown as percentage of infected plants on the total plants. The contamination of different treatments (blank; FG = wheat inoculated with *F. graminearum*; FG oat = uncontaminated wheat in proximity of oat kernels contaminated with *F. graminearum*) was shown at different stages of plant growth. In 2007 samples derived from eight plants for replicate, while in 2009 samples derived from 24 plants per replicate. Different letters within each group indicate statistically significant differences between treatments. Same letters within each group indicate not statistically significant differences between treatments. (**a**) 1st year experiment (2007); (**b**) 2nd year experiment (2009).

Data on the detection of *F. graminearum* in the plant portions at the different growth stages obtained from colony re-isolation are summarized in Figure 1. 

*Fusarium graminearum* colonies were detected in roots, crowns and stems of wheat plants but not in heads or kernels. Unfortunately, the blank treatment showed rare contaminations due to cross contamination in the room chamber; in particular, in 2007, all the portions of one plant (out of eight plants), and only the root of another plant at the second internode stage resulted contaminated, while only crown and stem of one plant reported contamination at the milky ripening stage; in 2009, only the crown of one plant (out of eight) was contaminated at the second internode stage. The treatment FG (wheat contaminated with *F. graminearum*) showed increasing levels of contamination by the time for each portion of plant in both the years (Figure 1): in 2007, the contamination of roots was 12.5% at shoots of 1–2 cm, 50% at second internode, and 62.5% at milky ripening; the crown contamination was of 62.5% at both first two stages (shoots of 1–2 cm and second internode) and 100% at the third stage; finally, the stems were not contaminated at shoots of 1–2 cm, while they had a contamination of 25% and 100%, at second internode and milk ripening, respectively; in 2009, crown contamination by *F. graminearum* resulted in 50% and 75% at second internode and milky ripening, respectively; stems were contaminated with 4% at second internode and 21% at milky ripening. The treatment FG oat was performed only in 2007. In general the level of *F. graminearum* contamination at the same plant growth stage, was higher in crowns than in stems . The heads and the kernelsanalyzed did not show any contamination by *F. graminearum*.

### 2.3. Qualitative PCR

All PCR data confirmed the morphological analyses and identified the fungal colonies isolated from the plants as *F. graminearum*.

### 2.4. Vegetative Compatibility Group

All the fungal colonies isolated were identified as *F. graminearum*. The identity as strain ITEM 126 was confirmed by VCG. In particular, we obtained *Nit*-M mutants that complemented each other from ITEM 126. All colonies generated *nit* mutants that were paired with *Nit*-M mutants of ITEM 126 and showed complementation in all pairings.

### 2.5. Quantitative PCR

**Figure 2:**
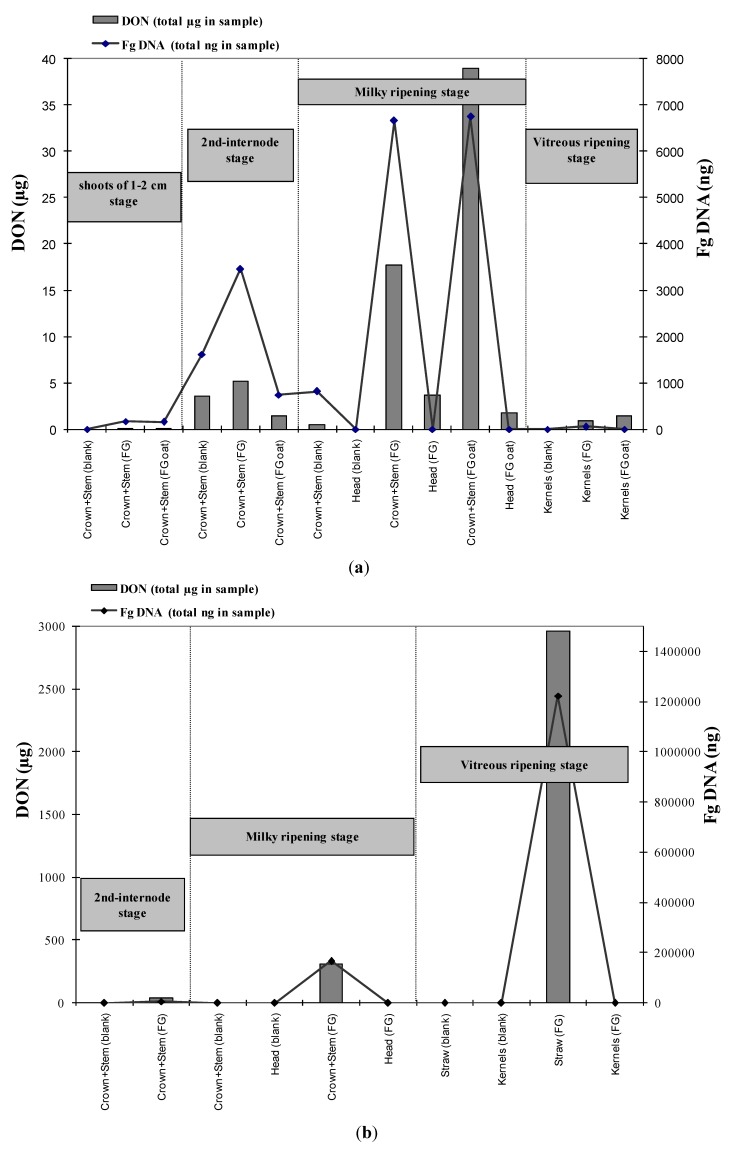
Combined data of quantitative PCR and deoxynivalenol (DON) occurrence evaluated on portions of the wheat plants of different treatments (blank; FG = wheat inoculated with *F. graminearum*; FG oat = uncontaminated wheat in proximity of oat kernels contaminated with *F. graminearum*), at different stages of plant growth. Data on DON are reported in total amount of mycotoxin in the sample analyzed. In 2007 samples derived from 8 plants for replicate, while in 2009 samples derived from 24 plants per replicate. (**a**) 1st year experiment (2007); (**b**) 2nd year experiment (2009).

The quantitative real-time PCR data are shown in Figure 2. *Fusarium graminearum* DNA was detected in roots, crowns and stems, at all the stages analyzed (Figure 2), while heads at the milky ripening stage and kernels at the vitreous ripening, were free from *F. graminearum* DNA. During the 2009 year of experiment, the straw analyzed at maturity, reported very high values of fungal DNA (9.8 ng DNA/mg plant tissue).

### 2.6. Chemical Analysis of DON and DON-3G

The results of analysis of DON content in plant materials, summarized in Table 1 (for the 2007 year of experiment) and Table 2 (for 2009), showed that heads and kernels were contaminated by DON at low level for all the treatments, except the controls that did not contain detectable levels of DON (treatment 1). Moreover the analyses of crowns and stems combined gave interesting results showing a high level of DON production *in planta*. The data showed that the production of DON in wheat plants started from the first stage of development (shoots of 1–2 cm). At the subsequent stages the level of DON increases (Table 1 and Table 2). Relevant is the occurrence of DON also in the upper portions of the wheat plants (heads, kernels and straw), with very high levels detected only in the straw (23.8 µg/g). The occurrence of the “masked DON” (DON-3G) at relevant levels, if compared to DON levels was detected at milky and vitreous ripening in crowns, stems and straw in 2009 (Table 2). The ratio DON-3G/DON was 0.3 in crown, 0.5 in stem and 0.7 in straw which may indicate an active role of the plant tissues in the glycosylation of DON following its production by *F. graminearum*. A very high value of DON-3G/DON ratio (23) was found in the head at milk ripening probably because most of DON translocated from the stem to the head is actively glycosylated by the plant and the absence of *F. graminearum* does not replace the glycosylated DON. Probably at the same time low amounts of both DON-3G and DON are passively translocated to the kernels where the DON-3G/DON ratio was found to be 1.2. Statistical analyses of DON-3G and DON was performed for straw and kernels collected at vitreous ripening in 2009. Mean level of DON was significantly higher than DON-3G in straw, whereas no statistically significant difference was observed for kernels (Table 2). No statistical analyses were performed for the other data, because the replicates were pooled in order to reach the minimum amount of plant material necessary for chemical analyses.

**Table 1:** Deoxynivalenol (DON) contamination, expressed in µg/g, in the first year of experiment (2007).

Stage of growth	Part of plant	Blank	FG	FG oat
Shoots of 1–2 cm	Crown + Stem	Nd	0.3	0.2
second internode	Crown + Stem	2.3	2.0	0.8
Milky ripening	Crown + Stem	0.2	14.6	27.0
	Heads	Nd	0.9	0.3
Vitreous ripening	Kernels	Nd	0.1	0.2

Notes: FG = wheat contaminated by *F. graminearum*; FG oat = wheat in proximity of oat kernels contaminated by *F. graminearum*; Nd = not detected (LOD = 0.02 µg/g for kernels, 0.05 µg/g for other plant materials).

**Table 2:** Deoxynivalenol (DON) and deoxynivalenol 3-glucoside (DON-3G) contamination, expressed in µg/g, in the second year of experiment (2009).

Stage of growth	Part of plant	Blank		*F. graminearum*	
		DON	DON-3G	DON	DON-3G
second internode	Crown	Nd	-	21.6	-
	Stem	Nd	-	4.6	-
Milky ripening	Crown	Nd	-	87.5	27.3
	Stem	Nd	-	10.3	5.5
	Heads	Nd	-	0.1	2.3
Vitreous ripening	Straw	Nd	-	23.8^a^	16.8^b^
	Kernels	Nd	-	0.1^a^	0.1^a^

Notes: Nd = not detected (LOD = 0.02 µg/g for kernels, 0.05 µg/g for other plant materials); - = not analyzed; Different letters in the row indicate statistically significant difference (P < 0.05) between DON and DON-3G; same letters in the row indicate no statistically significant difference (P > 0.05) between DON and DON-3G.

### 2.7. Relationship between Fungal and DON Contamination

**Figure 3:**
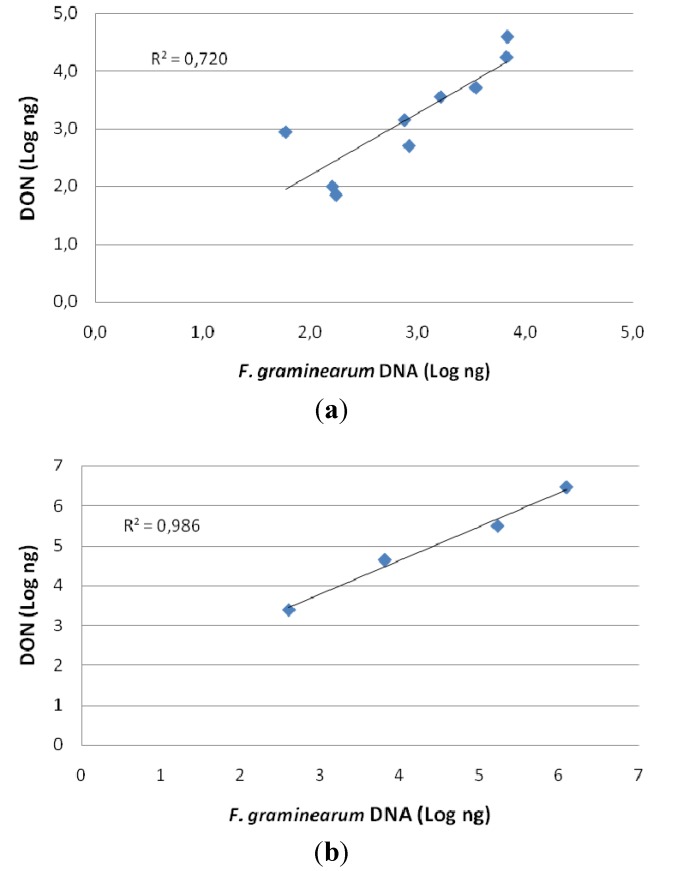
Correlation between *F. graminearum* quantity in plant material (detected by quantitative PCR) and DON content (detected by chemical analysis). The correlation coefficient is also shown for each graph. (**a**) First year of experiment (2007); (**b**) second year of experiment (2009).

The comparison of data derived from molecular detection of fungal occurrence by quantitative PCR and data of DON content by chemical analysis, are shown in Figure 2. The high values of correlation indexes, (*R*^2^ = 0.720 in 2007 and *R*^2^ = 0.986 in 2009) demonstrate a high correspondence between DON and fungal DNA (Figure 3). Heads and kernels, in which DON was detected in despite of fungal presence, were not considered in the calculation of these correlation indexes.

### 2.8. Discussion

The present study investigated the ability of a strain of *F. graminearum* to be transmitted from the seeds to the whole wheat plant, to colonize the wheat tissues and to produce DON *in planta*. Our findings demonstrated that the colonization of wheat crowns and stems through seeds inoculated with *F. graminearum* ITEM 126 conidial suspension occurred, but that the contamination could not be spread to the wheat heads and kernels. We used the highly sensitive molecular technique of quantitative real-time PCR to quantify possible small amounts of fungus not detected by traditional microscopical detection. Our results with molecular tools confirmed the microscopical results and exclude the presence of *F. graminearum* in the upper parts of wheat plants. *Fusarium graminearum* transmission from seeds to plants was previously reported in winter wheat by Duthie and Hall [9], who limited their study to the stems without analyzing the heads. On the other hand, studies investigating in wheat the *F. culmorum* or *F. graminearum* colonization process, from artificially inoculated seeds/crowns up to the heads, showed conflicting results [6,7,8,10,11]. Clement and Parry [10] demonstrated the presence of *F. culmorum* hyphae in the vascular system in part of wheat plants far from the point of inoculation. On the contrary, no evidence of hyphae of *F. graminearum* in the vascular cells was reported by Mudge *et al.* [7], suggesting a fungal growth at the interface of parenchyma with the lumen of the culm. Moreover, they reported the colonization of wheat heads and flag leaf nodes by *F. graminearum* after inoculation of the stem base of young seedlings. However, the possibility that insects (e.g., aphids) and/or mites can act as vectors of *Fusarium* spores from the stem base to the heads, must be considered [2]. The ability of *F. graminearum* to colonize wheat heads was demonstrated also by Poels *et al.* [8] that reported the migration of *F. graminearum* from the infected seeds to the heads. In accordance with Mudge *et al.* [7], Covarelli *et al.* [11] demonstrated that *F. culmorum* colonizes plant tissues by both intra- and intercellular growth, to explain the systemic growth through the stem. However, the colonization of heads by *F. culmorum* occurred only up to the third internode, being the last internode and heads free from any fungal colony. Similarly, in our study, *F. graminearum* colonies were not detected in the upper parts of the wheat plants: neither heads, nor kernels. A sort of barrier at head level seems to have been established by the wheat plants. However, if fungal aggressiveness of the selected strain, environmental conditions used in the experiments, and specific strain/variety combination are aspects that, alone or all together, could influence the plant ability to inhibit the head colonization, should be elucidated. To this respect, Beccari *et al.* [12] and Covarelli *et al.* [11] reported that *F. culmorum* caused symptoms of diseases in the whole plant in advance of the fungal colonization, suggesting a role of DON in inducing a host response far from the colonization points. On the contrary, Mudge *et al.* [7] reported that the occurrence of *F. graminearum* in the plants determined a suppression of the plant responses to the fungal invasion, with consequent absence of the FHB symptoms and allowing the final contamination of heads by the pathogen. Thus, further investigations are required to evaluate the mechanisms that allow the wheat plants to be free from the *F. graminearum* colonization of the heads originated from the seeds.

Several reports have shown the accumulation of DON in asymptomatic kernels caused by *F. graminearum* artificial inoculation of the heads at heading or anthesis [13,14]. However, this contamination has been always associated to the occurrence of fungal mycelium in the same heads. On the other hand, DON has been reported as commonly occurring in the wheat heads even when these are free from the colonization of *F. graminearum* or *F. culmorum* that were inoculated in the seeds or crowns [11,13,15]. This can be explained by the ability of DON to be water soluble [16] and to translocate through the stem tissues into the vascular system. Evidence for DON systemic translocation was confirmed by the occurrence of DON in xylem vessels and phloem sieve tubes [15]. In our work, the inoculation of wheat seeds by *F. gramineraum* led to the accumulation of DON in high amounts in the stems along all analyzed stages of plant growth. In the heads, little amount of DON was accumulated in milky stage, in absence of the fungus colonization, and this could be explained by DON translocation from the stems, where we detected it at high amounts. The high level of DON accumulation in the stems is worrisome not only for the possibility to be translocated in the heads, but also since DON, that is an inhibitor of protein synthesis, is accumulated in the plant tissues from the early stages of growth, affecting some of the physiological functions of the plants and reducing also its fitness and productivity. Moreover, Mudge *et al.* [7] hypothesized a role of DON in *F. graminearum* progression through the plant stem, although they considered it not essential for the infection. The high levels of DON-3G that we detected in the stems at two stages of the wheat plants (milky and vitreous ripening of heads) show a robust plant response to DON, confirming that DON plays an active role in the plant/*F. graminearum* interaction. The importance of DON in the *F. graminearum* colonization of wheat plant was also confirmed by Jansen *et al.* [17], that established that DON was not essential for initial fungal infection but it was required to suppress the production of host defense responses, such as the cell wall thickenings at the rachis node, during the colonization process. We detected in the heads, at both milky and vitreous ripening stages, higher amounts of DON-3G than DON, showing that the DON glycosylation activity of the wheat plants was very strong. Moreover, also dramatic higher levels of DON (and consequently of DON-3G) were detected in the straw respect to heads, confirming the data of Ludewig *et al.* [18], who reported lower content of DON in the kernels than rachis and/or straw. The authors speculated that this difference could be a consequence of a partial protection of kernels, due to their position in the heads, against a passive transfer of DON through the xylem by a reduced transpiration of kernel tissues compared to rachis and straw [18]. Moreover, Winter *et al.* [19] supposed the existence of a barrier zone at the interface between grain and rachilla, named “xylem discontinuity”, to explain the interruption of vascular transport of DON produced by *F. culmorum* to the kernels in wheat plants. This could explain the reduced DON amount in kernels compared rachis and peduncle, with a decreasing gradient of DON contamination from rachis, to straw and, finally, kernels [11,18,20,21]. To this respect, Ilgen *et al.* [22], and Gardiner *et al.* [23] reported that rachis tissues could be the site of DON-inducing synthesis factors, during the wheat heads infection by *F. graminearum*. However, in our work, although the analysis of the DON distribution in different part of the heads was not our goal, we could determine that DON occurrence was higher in straw compared the negligible amounts in the kernels, even in the absence of *F. graminearum* infection. If the heavy stem *F. graminearum* infection that we recorded just below the rachis, could play a role in stimulating the rachis inducing factors for DON synthesis, could be an interesting further investigation to carry out.

Wheat plants have been shown able to partially detoxify DON by a glycosylation process that transforms it in DON-3G [24,25,26]. We clearly showed that in both milky and vitreous kernel ripening stages, this role is actively played in different sites of the plants since the occurrence and levels of DON-3G and DON measured in stems, straw and kernels were comparable. Interestingly, we found much higher levels of DON-3G in heads as compared to DON at milk ripening with a ratio DON-3G/DON of 23. This result could be explained by the absence of *F. graminearum* in the head therefore most of the DON translocated from the stem is actively glycosylated in the head tissues. On the other hand, in wheat infected by *F. culmorum*, DON detoxification was supposed to occur mainly in the stem basis [19]. However, the very high DON-3G/DON ratio in heads as well as a ratio of 0.7 and 1.0 in straw and kernels, respectively, confirms that an active level of DON detoxification in the heads occurs. 

There is evidence that at flowering the wheat plants are more susceptible for the FHB attack and that this is the pathway for the *F. graminearum* contamination of heads and consequent final accumulation of DON in wheat grains. However, this is a result of several factors: genetic variability of *Fusarium* populations in the field, host response, environmental conditions and agronomic management, such as fungicide treatments at flowering, crop rotation, tillage, timing of harvest [1]. From our experiments, the infection of seeds by *F. graminearum* can participate to the final DON contamination of kernels at a very limited level. However, the ability of the fungus to be transmitted to the whole plant and to produce severe amounts of DON *in situ* affecting its fitness and productivity, requires a control of *F. graminearum* contamination at seeding. Therefore the control of the final DON amount in wheat kernels has not to be addressed toward a single factor influencing the FHB but requires a combined strategy including seed treatment. 

Finally the occurrence of DON-3G in the wheat kernels at the same levels of DON at maturity is a further reason of concern, since this metabolite can be hydrolyzed by humans once ingested, releasing DON [27]. Therefore, the total amount of DON and DON-3G should be considered in the evaluation of the risk derived from *F. graminearum* infection of wheat.

## 3. Experimental Section

This two year study, was carried out in a climatic chamber (phytotrone) with controlled conditions of temperature, and moisture. In the second year of experiments some modifications were made to improve the experiment design according to the results obtained in the first year.

### 3.1. Plant Material

The winter durum wheat cultivar Duilio, susceptible to the FHB and commercially important, was used for the experiments. An amount of 200 kernels from the sample selected for the seeding, was analyzed, in order to utilize seed kernels free from possible *Fusarium* contamination. The same sub-sample was analyzed for DON content by using HPLC (see below, chemical analysis).

In the whole experiment the plant material was analyzed at four different plant growth stages in the first year: (a) shoot of 1–2 cm; (b) second internode growth stage; (c) milky ripening of the kernels; (d) vitreous ripening of the kernels. The same growth stages, except shoot of 1–2 cm, were analyzed in the second year.

### 3.2. Experimental Trial for Fungal Inoculation

*Fusarium gramineraum* strain ITEM 126, obtained from the fungal collection of the Institute of Science of Food Production (ISPA) of the National Research Council (ITEM Collection [28]), was selected for the inoculation. The strain was tested for its capability to produce DON according with Quarta *et al.* [29]. The strain was grown on wheat kernels previously sterilized and placed at the dark and 25 °C for 3 weeks. 

**Figure 4:**
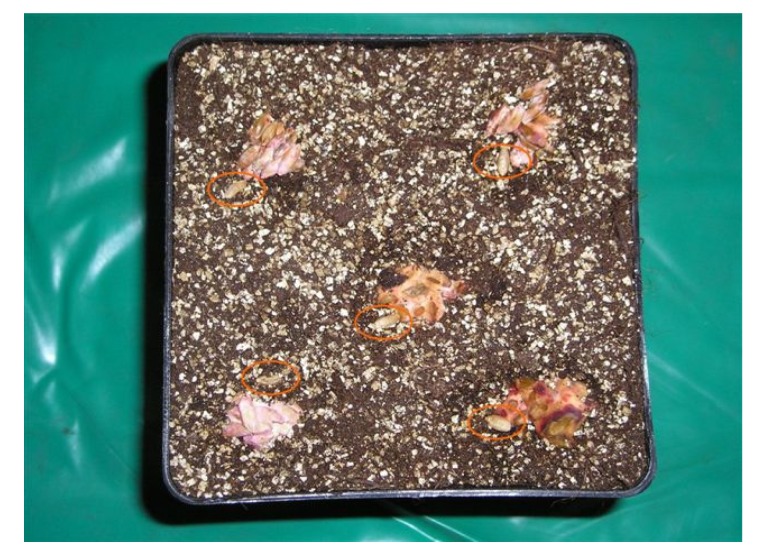
Oat kernels contaminated by *F. graminearum* and placed at the proximity of the seeds of wheat in the first year of experiment. The red circles indicate the wheat seeds.

In the 2007 experiment, two different techniques of artificial inoculation have been used: (a) direct spore suspension of *F. graminearum* strain on the kernels to be used as seeds; (b) contaminated oat kernels by the same strain of *F. graminearum* placed at the proximity of the seeds of wheat (Figure 4). Only the first kind of inoculation was used in the second year of experiment.

For each experiment, three treatments were analyzed in the first year: uncontaminated wheat (blank), wheat contaminated by *F. graminearum* (FG) and uncontaminated wheat in proximity of oat kernels contaminated by *F. graminearum* (FG oat). Only the blank and the FG treatments were analyzed in the second year of experiments. The spore suspension of ITEM 126, grown for one week on Salt Nutrient Agar (SNA) [30], was prepared with a concentration of 1 × 10^6^ conidia in sterile water. For the first kind of inoculation, the wheat seed kernels have been submerged in the conidial suspension and then dried under warm air. For the second kind of inoculation, through indirect contamination, oat kernels previously sterilized were inoculated by ITEM 126, grown for one week on SNA, and then placed at the dark at 25 °C for three weeks; aliquots of contaminated oat kernels were placed at the proximity of the wheat seeds in the pots (Figure 4). 

**Figure 5:**
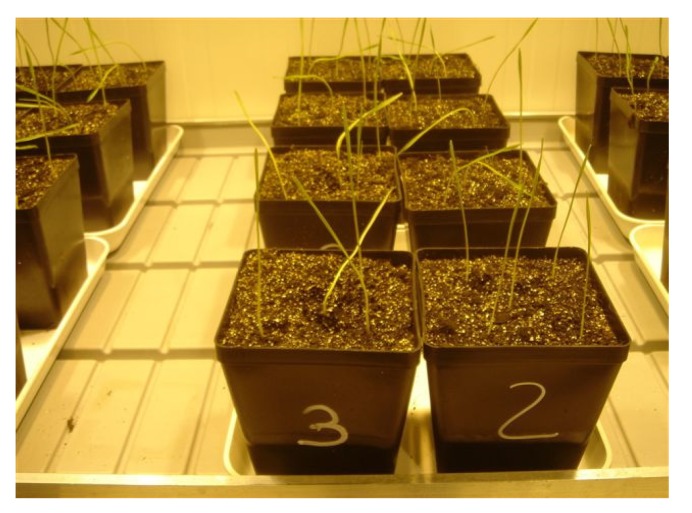
Pots in the climatic chamber at the emergence.

Seeds used for the blank treatment were suspended in sterile water and then dried under warm air. All the seeds were placed in pots containing a mixed medium (50:50 w/w) of soil (peat) and vermiculite free of *Fusarium* contamination as previously assessed. The pots were squared: 18 cm high, with a diameter of 52 cm at upper base and 42 cm at the lower base. After the seeding, the pots were placed in a climatic chamber (Figure 5) with controlled environmental conditions. Five seeds were placed for each pot and thinned out to two plants for each pot. The blank treatment had four replicates to be used for each plant growth stage to be analyzed. Each replicate had four pots to be considered as independent replicates; therefore, it was constituted by a total of eight plants that were all used for each analysis. The other two treatments had four replicates too in the first year of experiment, which was increased to 12 replicates (24 plants) in the second year. In order to reduce risks of cross contamination, the different treatments were accurately separated in the phytotrone.

### 3.3. Growth Chamber

We used as substrate for the germination/first development Compo Terraplant 2 (sphagnum enriched with fertilizers + 30% of sand) that has a sufficient amount of elements to ensure a good nutrition for the development of the plant; after jointing stage, we integrate with some UREA and ammonium nitrate to complete the cycle. A single irrigation was provided every morning for ensuring that the substrate was wet, but not saturated. The regulation of light, temperature, and humidity was performed as follows: the temperature was kept at 10–12 °C, and no light till emergence; from the emergence the temperature was at 4 °C, with 16 light hours/8 night, for a period from 1 to 5 weeks until vernalization was completed. After the vernalization, we maintained 16 light hours/8 night with growing temperature that gradually arrived at 25 °C and were kept until the end of the experiments. For all the cycle we set the growth chamber at 60%–70% humidity with ventilation.

### 3.4. Mycological Investigation by Microscopical and Molecular Analyses

Each plant produced two stems that were separated for the colony isolation from one stem and the molecular investigations on the second stem. Each analysis was destructive of all plants for each treatment (8 plants in 2007 and 24 plants in 2009).

*Isolation of fungal colonies.* In the first year, the whole plants were analyzed by cutting them in three parts: root, crown and stem, until the plants were at the second internode stage; at milky ripening also the heads were separately analyzed. Finally, at the vitreous ripening only the kernels were not analyzed. In the second year of experiment, root were no more analyzed, while at vitreous ripening also the straw was considered. The plant material was always superficially sterilized by using a 2% sodium hypochlorite solution, twice rinsed in sterile water and placed on a pentacholoronitrobenzene (PCNB) medium, semi-selective for *Fusarium* spp. Single-spore fungal cultures were obtained from the growing colonies re-isolated from the plants and identified morphologically as *F. graminearum*. 

*Vegetative Compatibility Group.* Each fungal colony isolated from the plants was scored for its Vegetative Compatibility Group (VCG), according to Leslie and Summerell [29] protocol, and compared against strain ITEM 126 (previously already analyzed for VCG), in order to confirm the identity of the strain used for the experiments. Mutants of *F. graminearum* ITEM 126 which were unable to use nitrate as a nitrogen source (nit) were obtained in MM + NaNO_3_ (0.2%) + KClO_3_ (3.0%). Mycelium plugs (5 mm) for each nit mutant were paired in Petri dishes containing MM + NaNO_3_ for vegetative complementation tests. Plates were incubated at 24 ± 2 °C during 7 to 21 days and then analyzed for heterokaryon formation.

*DNA extraction*. The plant material of the whole 8 or 24 part of plant (roots, crowns, stems, and heads) of each treatment were combined in each PCR analysis. Total DNA was extracted from freeze-dried ground plant material using “Genomic DNA from Plant-NucleoMag” DNA extraction kit (Macherey Nagel, Germany) according to the manufacturer’s instructions, but using a modification after lysing step: volume 250 µL of lysate was purified after addition of known amount of an exogenous amplifiable DNA (Internal Positive Control, Applied Biosystems, Foster City, USA), to evaluate the yield of the DNA extraction method and to normalize results.

*Qualitative PCR.* Specific primers for *F. graminearum* and PCR protocol by Nicholson *et al.* [31] were used for qualitative PCR. 

*TaqMan assays*. The quantification of ITEM 126 DNA in plant material was performed by real-time PCR. The primers and probe were derived after DNA sequencing of the about 400 bp fragment specific for *F. graminearum* [30] amplified in the strain ITEM 126. We designed the forward primer qGr2F (5’-CAGGTGGAAACAGATGACAAGATT-3’) and the TaqMan probe qGrprobe (5’-AGGCACATATAACTGCACTT-3’), able to amplify a unique 72 bp fragment in combination with the reverse primer Fg16R [31]. *F. graminearum* quantitative real-time assay was carried out by using an ABI PRISM 7000 system (Applied Biosystems). The PCR reaction mixture composition was: 1× TaqMan Master Mix (Applied Biosystems), 300 nM qGr2F and Fg16R primers, 1 μL (out of 100 μL) of total DNA as template, 200 nM 6-FAM-labeled qGrprobe, 1× Exogenous IPC Mix (containing optimized primers and VIC-labeled probe), and deionized water to a final volume of 25 μL. The temperature profiles in the ABI PRISM 7000 system were as follows: 2 min at 50 °C for uracil-Nglycosidase (UNG) enzyme activity, 10 min 95 °C to denature the UNG enzyme and to activate the polymerase, 40 cycles at 95 °C for 15 s and 60 °C for 1min. TaqMan reactions were performed in triplicates for the 2 sub-samples of each plant sample in MicroAmp 96-well plates. In each real-time PCR experiment, a serial dilution of *F. graminearum* DNA (ITEM 126) (ranging from 6 ng to 6 × 10^−5^ ng) and Exogenous IPC DNA (ranging from 4× to 0.5×) were included as standards.

*Chemical analysis of DON and DON-3G.* Chemical analysis of DON was carried out on crowns and stems of the shoot of 1–2 cm, second inter-node and milky ripening growth stages, heads only for the milky ripening growth stage, and kernels only at the vitreous ripening. All samples collected in 2009 were also analyzed for DON-3G but the second inter-node samples. The stems and crowns of the total plants of each replicate were combined at each stage since the amount of plant material available for the chemical extraction was too low if analyzed separately. Samples of straw and kernels collected at vitreous ripening in 2009, were analyzed for DON and DON-3G in triplicates due to the higher number of plants cultivated (24 instead of 8 plants). The analyses were performed by HPLC/UV/DAD with a previous purification of crude extract on immuno-affinity column following the method of MacDonald *et al.* [32] that was developed and collaboratively validated for the determination of DON in cereals and derived products. The method was applicable to the samples analyzed in this study for the determination of DON and DON-3G. The use of DONPREP^®^ immuno-affinity column (R-Biopharm, Darmstadt, Germany) permitted the determination of both DON and DON-3G due to its cross reactivity for the two toxins. Precision and accuracy experiments for DON and DON-3G determination in wheat plant materials gave mean recoveries of 80%–85% and mean repeatability of results of 7%–10%. The detection limits were of 0.05 µg/g for crowns, stems and heads and 0.02 µg/g for the kernels.

### 3.5. Statistical Analyses

Statistical analyses were performed by using the GraphPad Instat software (Instat, San Diego, CA, USA). Data were subjected to the paired t test (one tailed P value). Values were judged to be significantly different if P values were <0.05.

## 4. Conclusions

In conclusion, from the data of our experiments, we can conclude that:

- *Fusarium graminearum* can grown systemically in the plant tissues from the seeds. However, there is a barrier at the head level for the fungus that gets the heads free from the fungus;
- the level of contamination *in planta* of both *F. graminearum* and DON has been shown extremely high;
- low levels of DON and DON-3G, equivalent to 0.2% of total DON produced by *F. graminearum* in the plant, were found in the kernels that resulted free of *F. graminearum*;
- DON and DON-3G can be moved from lower parts of the plants to the heads probably due to their water solubility;
- DON is largely glycosylated to DON-3G by the whole wheat plant at different growth stages, leading to a high DON-3G accumulation in different parts of plants;
- a good correlation index between the DNA of *F. graminearum* detected in the samples by quantitative PCR and DON content has been obtained;
- the high level of DON occurring in the straw, detected in this study, is extremely worrisome since straw is used for bedding and this is consumed by livestock and therefore is an additional source of mycotoxin.
